# Nickel(II)-Based Building Blocks with Schiff Base Derivatives: Experimental Insights and DFT Calculations †

**DOI:** 10.3390/molecules26175316

**Published:** 2021-09-01

**Authors:** Néstor Novoa, Carolina Manzur, Thierry Roisnel, Samia Kahlal, Jean-Yves Saillard, David Carrillo, Jean-René Hamon

**Affiliations:** 1 Laboratorio de Química Inorgánica y Organometálica, Departamento de Química Analítica e Inorgánica, Facultad de Ciencias Químicas, Universidad de Concepción, Edmundo Larenas 129, Casilla 160-C, Concepción, Chile; 2 Laboratorio de Química Inorgánica, Instituto de Química, Facultad de Ciencias, Pontificia Universidad Católica de Valparaíso, Avenida Universidad 330, Curauma 2371985, Valparaíso, Chile; cecilia.manzur@pucv.cl; 3 Univ Rennes, CNRS, ISCR (Institut des Sciences Chimiques de Rennes)–UMR 6226, F-35000 Rennes, France; thierry.roisnel@univ-rennes1.fr (T.R.); samia.kahlal@univ-rennes1.fr (S.K.); jean-yves.saillard@univ-rennes1.fr (J.-Y.S.)

**Keywords:** nickel, Schiff base complexes, Sonogashira cross coupling, X-ray crystal structure, DFT calculations

## Abstract

We have recently reported a series of neutral square planar tridentate Schiff base (L) complexes of the general formula [(L)M(py)], showing relatively high first-order hyperpolarizabilities and NLO redox switching behavior. In the present study, new members of this family of compounds have been prepared with the objective to investigate their potential as building blocks in the on-demand construction of D-π-A push–pull systems. Namely, ternary nickel(II) building blocks of general formula [(L^A/D^)Ni(4-pyX)] (**4–7**), where L^A/D^ stands for an electron accepting or donating dianionic O,N,O-tridentate Schiff base ligand resulting from the monocondensation of 2-aminophenol or its 4-substituted nitro derivative and β-diketones R-C(=O)CH_2_C(=O)CH_3_ (R = methyl, anisyl, ferrocenyl), and 4-pyX is 4-iodopyridine or 4-ethynylpyridine, were synthesized and isolated in 60–78% yields. Unexpectedly, the Sonogashira cross-coupling reaction between the 4-iodopyridine derivative **6** and 4-ethynylpyridine led to the formation of the bis(4-pyridyl) acetylene bridged centrosymmetric dimer [{(L^D^)Ni}_2_(µ^2^-py-C≡C-py)] (**8**). Complexes **4–8** were characterized by elemental analysis, FT-IR and NMR spectroscopy, single crystal X-ray diffraction and computational methods. In each compound, the four-coordinate Ni(II) metal ion adopts a square planar geometry with two nitrogen and two oxygen atoms as donors occupying trans positions. In **8**, the Ni…Ni separation is of 13.62(14) Å. Experimental results were proved and explained theoretically exploiting Density Functional Theory calculations.

## 1. Introduction

Schiff bases, characterized by an imine or azomethine group (>C=N- or –C(H)=N-, respectively), can be easily synthesized in one step upon condensation of an active carbonyl compound with a primary amine and elimination of one water molecule [1]. The use of differently substituted aldehydes or ketones and amines counterparts, most of the time commercially available, allows us to adjust their geometric and electronic properties and the formation of complex and sophisticated structures [2,3]. Schiff bases usually coordinate to a metallic ion through the imine nitrogen atom and are able to complex most metals in the periodic table with distinct oxidation states, coordination numbers and geometries [4,5,6,7], thus finding a plethora of applications within numerous scientific areas [8,9]. Their diverse applications, associated with their structural flexibility, made them essential compounds that have extensively been used in homogenous and heterogeneous catalysis [10,11,12,13,14], chemical sensing [15,16,17,18], nonlinear optics (NLO) [19,20,21,22], beside remarkable pharmacological and biological activities [23,24,25,26,27,28], to name a few.

Although the tetradentate chelating Schiff base ligands with the dianionic N_2_O_2_ donor set, known as salen-like ligands [29,30], and their corresponding complexes continue to be the most popular and the most studied for their numerous applications (sensing [31], medicine [32,33], catalysis [14,32,34], as synthons for various supramolecular structures [35,36,37]), Schiff base ligand with lower denticity, namely tridentate, are also common [30,38]. Tridentate ligands offer three donor atoms (mostly O, N and S) and act as anionic pincer-type ligand to efficiently chelate the metal ions, thus generating versatile conformationally rigid [M(L^A/D^)]^n+^-type metalloligands, where L^A/D^ represents electron withdrawing (A) or electron donating (D) tridentate Schiff base ligands. Implementation of the remaining vacant coordination sites on the metal centers with additional co-ligands allows us to construct new multimetallic architectures with desired properties [39,40]. Using 4,4’-bipyridine (4,4’-bipy) as co-ligand does generally lead to the formation of centrosymmetric dinuclear species constructed from [(L^A/D^)M]^n+^ units which are connected through the bidentate 4,4’-bipy ligand having nitrogen-donor atoms [41,42,43,44]. Mononuclear complexes in which the 4,4’-bipy ligand shows monodentate coordination mode are rare and difficult to prepare [43]. This, therefore, strongly limits their use in the construction of asymmetric species and especially D-π-A push–pull systems with potential second-order nonlinear optical (NLO) response [45]. A convenient tool to assemble donor (D) and acceptor (A) metalloligands bridged by 4,4’-bipy or one of its conjugated derivatives into a NLO-active assembly would, therefore, consist in the linkage of two building blocks containing appropriately functionalized pyridine co-ligands via “click” chemistry [46], Schiff condensation [1,2] or carbon–carbon cross coupling reactions [47,48].

**Scheme 1:**
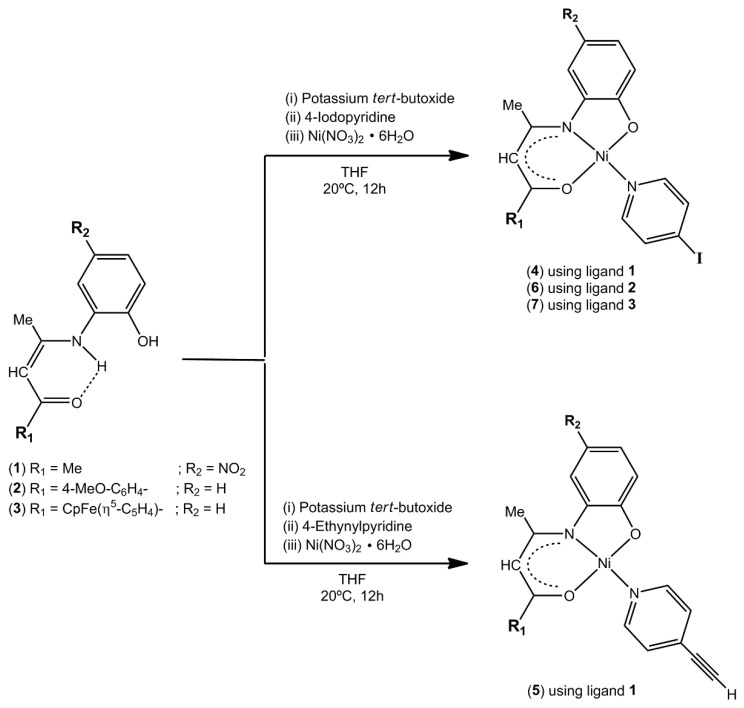
Synthesis of complexes **4**–**7**.

**Scheme 2:**
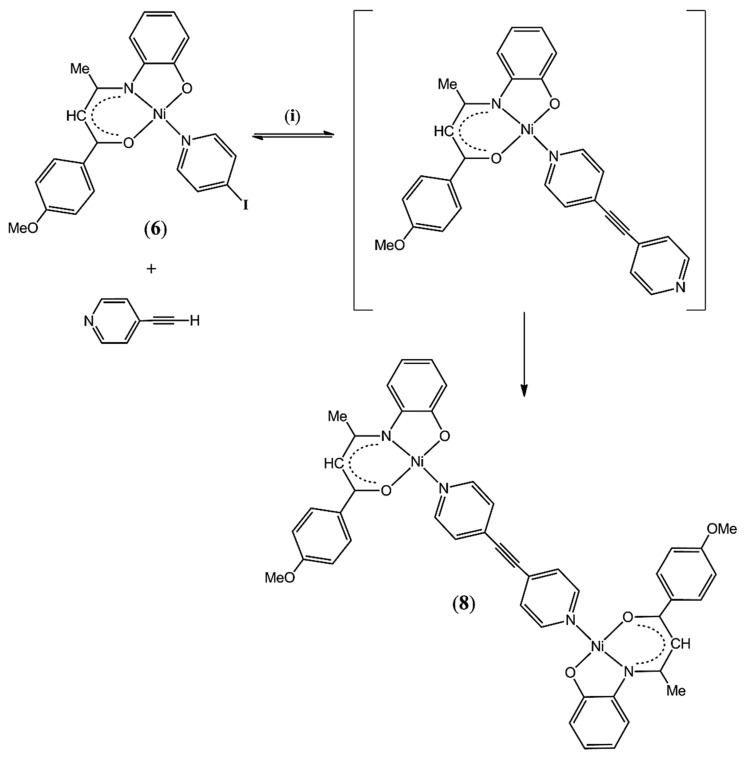
Formation of the binuclear complex **8**; (i) = *trans*-PdCl_2_(PPh_3_)_2_, CuI, TEA:THF (1:5, *v*:*v*), 12 h at 20 °C.

We have recently developed the chemistry of two new series of neutral square planar four-coordinate nickel(II)- and copper(II)-tridentate Schiff base (L) complexes of the type [(L)M(py)], where py represents a pyridine-based co-ligand [49,50,51]. Interestingly, those ternary chromophores exhibit relatively high first-order hyperpolarizabilities and show NLO redox switching behavior in the specific case of the electroactive pyridiylmethylenepyran co-ligand [51]. As an extension of this previous work, in this contribution we designed and investigated a new family of nickel(II) based ternary building blocks of general formula [(L^A/D^)Ni(4-pyX)] where L^A/D^ stands for a dianionic O,N,O-tridentate Schiff base ligand resulting from the monocondensation of 2-aminophenol or its 4-substituted nitro derivative and β-diketones: R-C(=O)CH_2_C(=O)CH_3_ (R = methyl, anisyl, ferrocenyl), and 4-pyX is 4-iodopyridine or 4-ethynylpyridine (see formulas of complexes **4–7** in Scheme 1), with the aim of exploring their potential ability to generate D-π-A type assemblies via Sonogashira cross-coupling reactions [48]. Herein, we report on the synthesis, analytical and spectral characterization of the mono- **4–6** and bi-metallic **7** building units, and of the unexpected dipyridylethyne-bridged centrosymmetric dimer **8** (see Scheme 2), as well as the crystal and molecular structures of compounds **4–6** and **8**. Density functional theory (DFT) calculations at the PBE0/Def2-TZVP level performed on all these complexes shed light on their electronic structure and bonding.

## 2. Results and Discussion

### 2.1. Synthesis and Characterization 

The ternary neutral mononuclear complexes **4–6** and the heterobimetallic species **7** were synthesized following a simple one-pot three-step templated reaction [49]. First, the corresponding diprotic unsymmetrical Schiff base proligands **1–3** were doubly deprotonated with a slight excess of potassium *tert*-butoxide in THF at ambient temperature. A stochiometric amount of 4-iodopyridine or 4-ethynylpyridine hydrochloride and trimethylamine (TEA) was then added to the dark red reaction mixture before dropwise addition of a THF solution of nickel(II) nitrate hexahydrate salt (Scheme 1). Complexes **4–7** are isolated in reasonably good 60–78% yields as brown (**4**, **5**) and red (**6**, **7**) powders. They are all thermally stable, as well as air and moisture insensitive on storage under ordinary conditions. The four compounds exhibit good solubility in common polar organic solvents but are not soluble in ethanol, diethyl ether and hydrocarbons as pentane and hexane.

As a first attempt in the construction of donor–acceptor type complexes starting from the as-prepared building blocks **4–7**, we reacted the electron releasing derivative **6** with a stoichiometric amount of 4-ethynylpyridine hydrochloride under C-C Sonogashira cross-coupling reaction conditions (see Section 3.2.5 for details). To our surprise, the reaction results in the formation of dimeric species **8** in which the two Schiff base nickel(II) metalloligands are linked through the bis(4-pyridyl) acetylene moiety, instead of the expected unsymmetrical mononuclear derivative [(4-MeO-C_6_H_4_-C(O)CH=C(CH_3_)N-C_6_H_4_-2-O)Ni(NC_5_H_4_-C≡C-C_5_H_4_N)] (Scheme 2). Complex **8** was isolated as a bench-stable dark red powder in 43% yield, poorly soluble in common organic solvents. Interestingly, a few black crystals of the known Pd(II) complex *trans*-PdI_2_(PPh_3_)_2_ (**9**) [52] deposited from the washings and were collected by filtration.

The formation of the dimeric species **8** could be explained by a two-step process (Scheme 2). Firstly, based on the formation of the diiodo palladium complex **9**, C-C cross-coupling reaction between the building block **6** and the in situ generated 4-ethynylpyridine takes place to form the mononuclear intermediate in which the bis(4-pyridyl) acetylene is bound to the Ni(II) center in a monodentate coordination mode. Then, in a second step faster than the first one, the mononuclear intermediate would react via ligand exchange with yet unreacted complex **6** to form the final product **8**. Another possibility could be the dissociation of complex **6** under the catalytic conditions to generate a solvated nickel species and free 4-iodopyridine that would undergo Pd-catalyzed C-C coupling with 4-ethynylpyridine to form the bis(4-pyridyl) acetylene that is ultimately captured by two metalloligands to form **8**. However, this latter dissociation/association process seems more unlikely as the building block **6** is stable under the employed experimental conditions, i.e., THF and base (see Section 3.2).

The bulk purity of all the new compounds **4–8** was determined through elemental analysis. Their composition and formulation were established from FT-IR and ^1^H NMR spectroscopy, and single-crystal X-ray diffraction analysis. The solid-state FT-IR spectra exhibited the characteristic strong intensity bands in the ranges 1610–1550 cm^−1^, due to the *ν*(C^···^C), *ν*(C^···^N), *ν*(C^···^O) stretching vibrations of the Schiff base ligand, suggesting its ONO- tridentate coordination mode to the Ni(II) metal ion [49,50,51]. More specifically, the symmetrical N-O stretching mode of the nitro group was observed at 1300 and 1310 cm^−1^ for **4** and **5**, respectively [53]. Moreover, the vibration attributed to *ν*(C≡C) is seen at 2227 cm^−1^ in the spectrum of **5** and at 2111 cm^−1^ in that of **8** (Figure S1, Supplementary Materials). For all the compounds, the strong deformation modes of the C-H bonds appeared about 760 cm^−1^.

**Figure 1:**
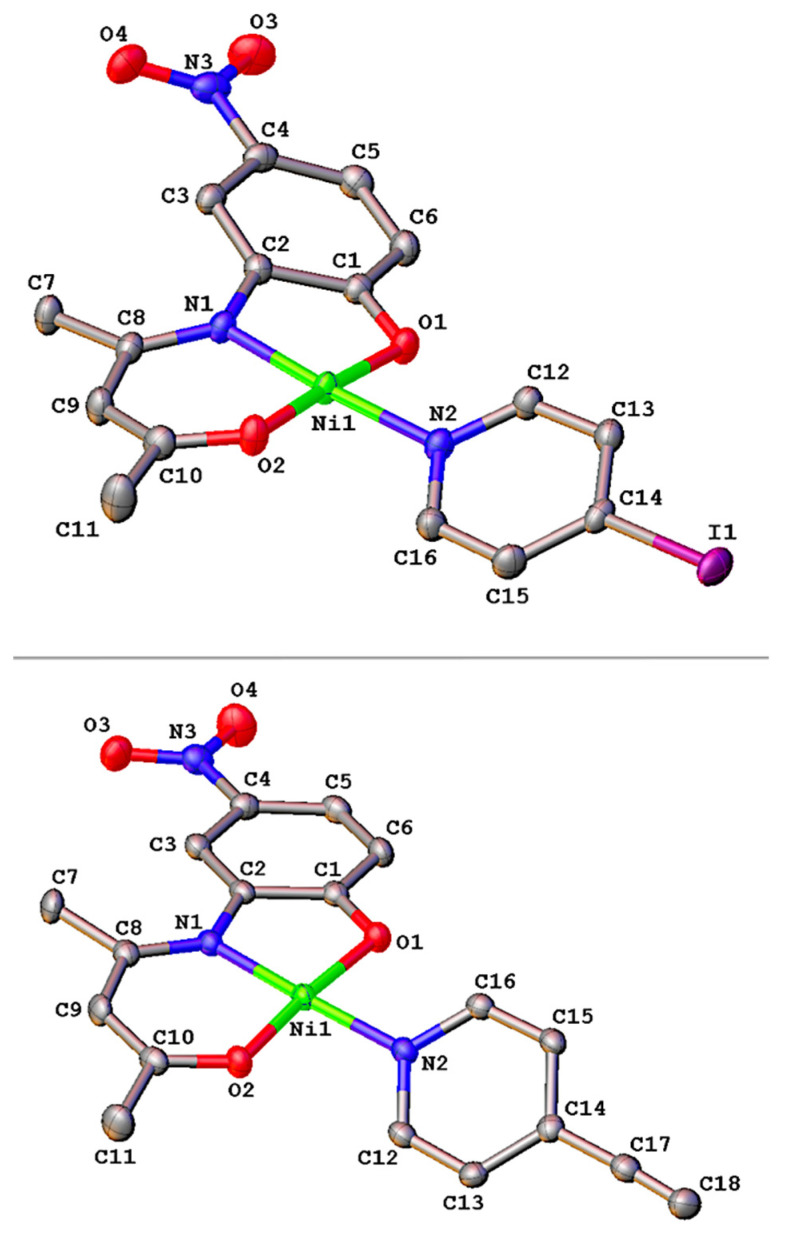
Molecular structures of complexes **4** (**top**) and **5** (**bottom**), with labelling scheme for selected atoms. Hydrogens have been omitted for clarity. Thermal ellipsoids are drawn at the 50% probability level.

The ^1^H NMR spectra displayed the expected resonance patterns consistent with the proposed structures. Full assignments are provided in Section 3.2 and the spectra of complexes **7** and **8** are depicted in Figures S2 and S3, respectively, as representative examples. In each spectrum, the singlets observed in the 2.42–2.59 ppm and 5.27–5.93 ppm ranges are due to the methyl (C7, Figure 1) and pseudo-aromatic CH=C methine protons (H-9), respectively. Singlets assigned to the methyl (C11, Figure 1), methoxy and free cyclopentadienyl ring protons are also seen at 1.84, 1.85, 3.72, 3.83 and 4.06 ppm for **4**, **5**, **6**, **8** and **7**, respectively. The magnetically nonequivalent aromatic protons of the imino-phenoxo fragment give rise to three (in **4** and **5**) and four (in **6**, **7** and **8**) signals in the 6.31–8.13 ppm range with integral ratios of 1:1:1 and 1:1:1:1, respectively. The presence of the 4-substituted pyridine co-ligand was confirmed by the observation of a pair of low-field broad resonances integrating each for 2 H. The *ortho*-protons appear as the most downfield shifted signals of the spectra [49,51]. 

With the aim to explore the absorption spectrum of the new carbon-carbon cross coupling derivative **8**, we carried out the UV-vis spectrum of the complex in CH_2_Cl_2_ solution. As expected, the spectrum is similar to that previously reported for its 4,4’-bipy bridged counterpart [44], exhibiting the same set of three absorption bands(Table S1). Accordingly, the high-energy band (a) observed at 295 nm can be attributed to the metal-to-ligand charge transfer transitions (MLCT) with a minor contribution of a π→π* transition. Band (b) that shows up at 417 nm can be assigned to a LMCT + π→π* transitions, whereas the low-energy absorption band (c), appearing at 456 nm, can be mainly attributed to a π→π* electronic transition.

### 2.2. Description of Crystal Structures

**Figure 2:**
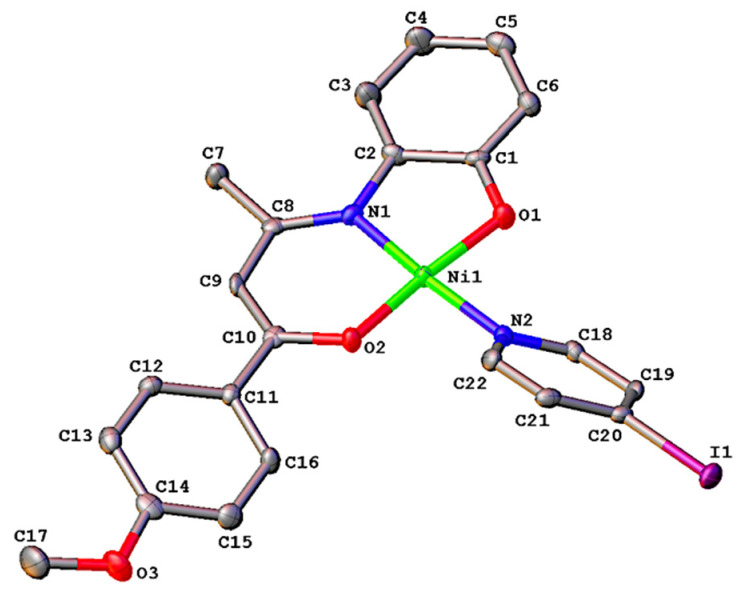
Molecular structure of complex **6** with labelling scheme for selected atoms. Hydrogens have been omitted for clarity. Thermal ellipsoids are drawn at the 50% probability level.

**Figure 3:**
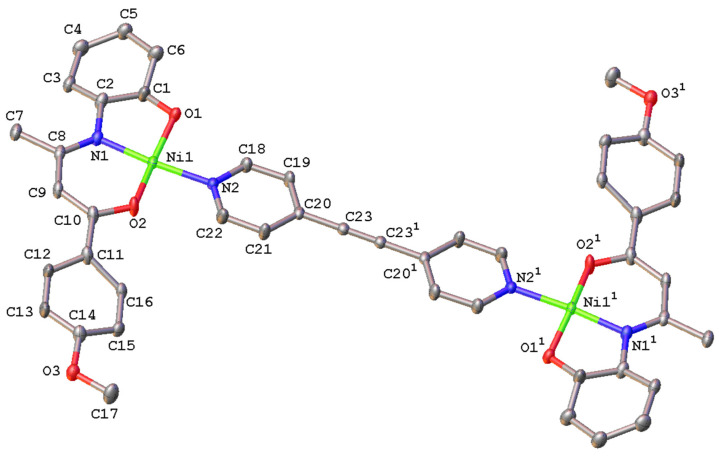
Molecular structure of complex **8** (molecule **A**)with labelling scheme for selected atoms. Hydrogens have been omitted for clarity. Thermal ellipsoids are drawn at the 40% probability level. Half unit of molecule was generated by symmetry operations -x, -y, -z.

Diffraction-quality single crystals for X-ray structure investigation were obtained for compounds **4**, **5**, **6** and **8** by slow evaporation of the solvent from a saturated solution of the compound (see Section 3.2 and Section 3.3 for details), while single crystals of **9** formed by crystallization from a methanol/dichloromethane mixture. The crystal structure of **9** which has already been published as its bis-chloroform solvate [52], will not be discussed here. Perspective views of the nickel(II) complexes (**4–6** and **8**) are shown in Figure 1, Figure 2 and Figure 3, and that of the Pd(II) derivative **9** is displayed in Figure S4. Bond distances and angles for the first Ni(II) coordination sphere are gathered in Table 1, and selected bond distances and angles of complexes **4**–**6** and **8** are listed in Table S2. The single-crystal X-ray diffraction studies confirm the ternary nature of complexes **4**, **5**, **6** and **8** with the ONO-tridentate Schiff base framework and the 4-substituted pyridine ligand coordinated to the central Ni(II) ion. In each case, the four-coordinate metal ion is bonded to a N_2_O_2_ donor atoms set. The Ni(II) center adopts a quasi-perfect square planar geometry in the mononuclear species **4–6** that is very slightly distorted in the bimetallic counterpart **8**. This is evidenced by the rather similar values of the four-coordinate geometry *τ*_4_ index of 0.054, 0.066 and 0.066 determined for **4**, **5** and **6**, respectively, whereas it is almost two-fold greater for **8** (*τ*_4_ = 0.124; *τ*_4_ = 0 for a perfect square planar geometry [54]. In the four complexes, the O-C, C-C and C-N bonds falling between single and double bond lengths and bond angles of *sp^2^* hybridized atoms (Table S2) [55], suggests a significant electron delocalization throughout the entire Schiff base ligand. The other aspects of the structures are discussed below in three sections for the sake of simplicity.

Complex **4** crystallizes in the monoclinic centrosymmetric space group P2_1_/c while complex **5** crystallizes in the orthorhombic centrosymmetric space group Pcnb, with in each case one molecule in the asymmetric unit. Both compounds consist of a common electron withdrawing dianionic Schiff base ligand chelating a nickel(II) ion through a deprotonated amide nitrogen atom (N1) and the carbonyl (O2) and phenolato (O1) oxygen atoms. The fourth position of the metal coordination sphere is occupied by the nitrogen atom (N2) of the 4-iodopyridine in **4** and of the 4-ethynylpyridine in **5** (Figure 1). In the square planar environment of the Ni(II) center, the four donor atoms adopt a N_2_O_2_ *trans*-configuration with O-Ni-O and N-Ni-N diagonal angles barely deviating from linearity (~175°, see Table 1). The Ni-O and Ni-N bond distances of complexes **4** and **5** span the range 1.817(2)–1.9402(16) Å (Table 1). They are typical of ternary square-planar Ni(II) Schiff base complexes [49,51]. In **5**, a classical bond distance of 1.159(4) Å is measured for the terminal ethynyl C17-C18 triple bond, which is linear with a C14-C17≡C18 angle of 177.1(3)° [55]. In both complexes, the fused five- and six-membered heterometallacycles are essentially co-planar, making torsion angles of 12.6(9)° and 3.7(7)°, respectively, around the Ni-N1 axis. On the other hand, the 4-substituted pyridine co-ligand is twisted by 31.4(8)° and 35.1(6)° with respect to the metal coordination plane. In both **4** and **5**, the electron withdrawing nitro group is coplanar with the phenyl ring, making dihedral angles of 3.8(4)° and 3.6(2)°, respectively. This suggests a significant delocalization of the electron density throughout the entire π-conjugated system. Lastly, the packing of both complexes **4** and **5** shows that the structures are stabilized by a network of intermolecular hydrogen bonds leading to the formation of a dipolar stacked orientation for **4** and zig-zag infinite chains for **5** (Figures S5 and S6, and Table S3).

The mononuclear nickel(II) derivative **6** crystallizes in the orthorhombic centrosymmetric space group Pbca with one molecule in the asymmetric unit. As in related complexes **4** and **5** above, the coordination sphere of the centered Ni(II) ion is made up of the ONO-tridentate Schiff base and 4-iodopyridine ligands (Figure 2). The nickel center adopts a square planar geometry with the nitrogen and oxygen atoms occupying mutually *trans* dispositions with diagonal angles of 174° (Table 1). The bond lengths and angles involving the metal center (Table 1) are very similar to those measured for its parent complex [49], the Ni-N(2) bond length always being the larger one. The fused five- and six-membered heterometallacycles are co-planar (2.7(2)°). The anisyl substituent makes a dihedral angle of 20.7(2)° with the plane of its attached heterometallacycle. The pyridine ring is twisted by 38.5(2)° with respect to the metal coordination plane. As expected, those metrical data are similar to those reported for four-coordinate Ni(II) Schiff base complexes containing anisyl group and 4-substituted pyridine co-ligand [49]. The molecular packing analysis of **6** reveals alternating spirals along the a-axis direction (Figure S7). Interestingly, no strong H-bond or π-π interactions can be found, thus attributing the crystal stability to weak supramolecular interactions.

The binuclear complex **8** crystalizes in the monoclinic centrosymmetric P2_1_/c space group with half a molecule in the asymmetric unit. The two halves of the dimeric entity are related by a crystallographic inversion center in the middle of the C≡C triple bond of the bridging bis(4-pyridyl) acetylene ligand, with a Ni^…^Ni separation of 13.6186(14) Å. The Ni(II) ion is tetracoordinated and lies in a slightly distorted square-planar environment (*τ*_4_ = 0.124), with the coordination sphere formed, such as in **6** above, by the ONO donor set of the anisyl-containing Schiff base ligand and the N-pyridyl atom of the conjugated bis(4-pyridyl) acetylene spacer (Figure 3). The three bond lengths involving the Ni(II) center and the donor atoms of the pincer-type ligand are similar to those found in the mononuclear counterpart **6** (Table 1). The Ni(1)-N(2) bond distance is somewhat larger by 0.04 Å in the dimeric species as previously noted with the corresponding 4,4’-bipyridine-bridged derivative [44]. Although the N(1)-Ni-N(2) transoid angle is of the same order as those found in **4–6**, the O1-Ni-O2 angle averages 167.5° (Table 1), significantly deviating from linearity. Compared to complex **6**, each half unit of the dimeric derivative **8** is rather flat, the anisyl group and the pyridyl ring being slightly twisted by 12.1(2)° and 9.9(2)°, respectively. Interestingly, the double coordination of the bis(4-pyridyl) acetylene by two [Ni(L^D^)] metalloligands does not affect the metrical parameters of the conjugated linker. The C≡C bond length of 1.199(6) Å as well as the ring (mean plane)-C≡C-Ring (mean plane) twist angle of 0.0(5)° are identical to those measured in free 4,4’-dipyridylacetylene (1.199(3) Å and 0.0(2)°, respectively) [56]. A similar C≡C bond length (1.184(11) Å) and ring twist angle (0.6(5)°) have also been reported for a related py-C≡C-py spaced dimeric Fe(II) complex featuring N_2_O_2_-tetradentate Schiff base-like ligand [56]. Finally, the crystal packing is stabilized by hydrogen bonds between O(3) and the -C_7_H_3_ and N1/O2 fragments of neighboring molecules to give a ladder-type packing oriented along the b-axis (Figure S8, Table S3).

### 2.3. DFT Analysis

**Figure 4:**
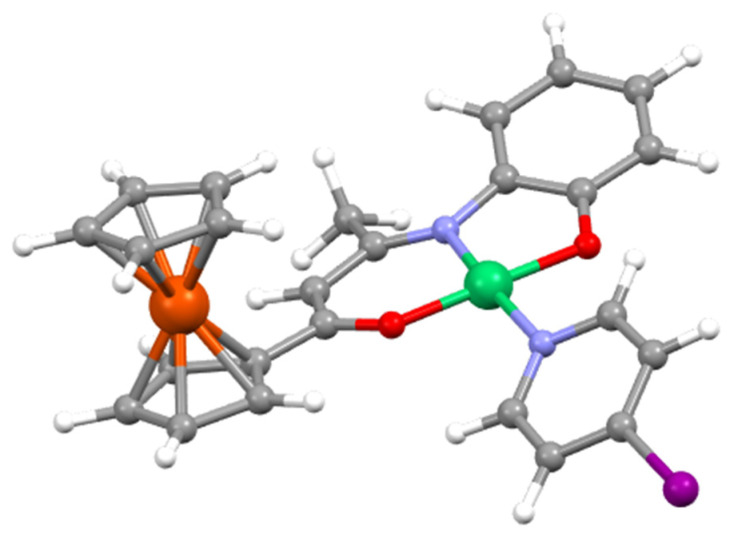
The DFT-optimized structure of complex **7**.

The DFT-optimized geometries of complexes **4**, **5** and **6** (Figure S9) are in a good agreement with their experimental X-ray counterparts. Selected optimized metrical data associated with the nickel coordination sphere are given in Table 1. There are similar to their corresponding X-ray values and show even a better homogeneity. The associated Wiberg bond indices are also quite homogeneous. The values computed for **7**, for which no X-ray structure is available, fit well into the series. Its optimized structure is provided in Figure 4.

The (moderate) rotation of the pyridine ring away from coplanarity with the Ni coordination plane is also reproduced in complexes **4**–**6**, indicating it is not a solid-state packing effect, but the result of intra-molecular steric effects. The computed natural atomic orbital (NAO) charges remain almost the same for the four complexes within the Ni coordination sphere (Table 2). Thus the various substituents on the pyridine and O,N,O-tridentate Schiff base ligands have little effect on the Ni coordination sphere.

**Figure 5:**
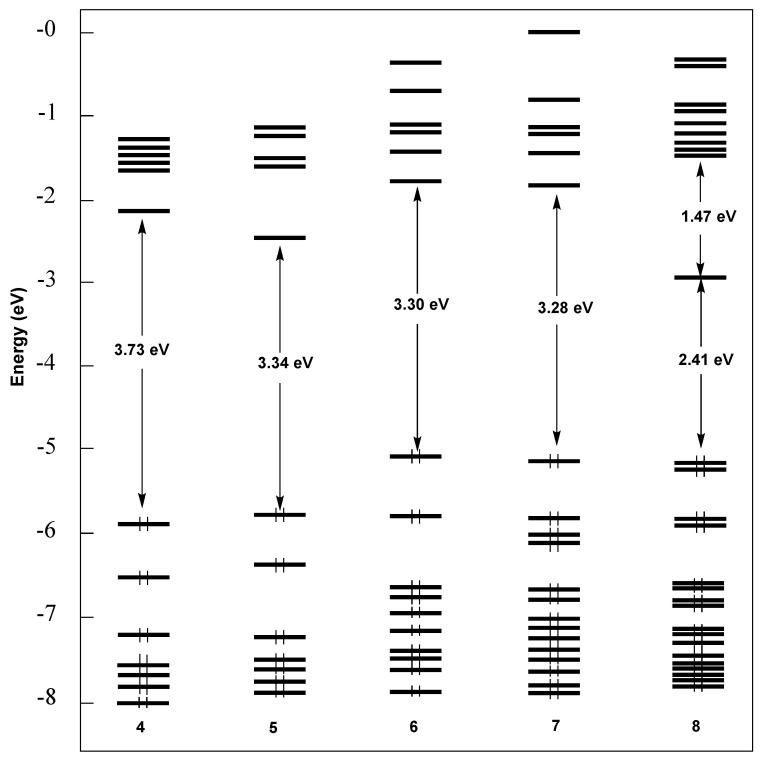
The MO energy level ordering of the five investigated complexes.

They however perturb differently the overall electronic structure of the four complexes through their specific action on the ligands they are attached to. This is exemplified by the frontier Kohn–Sham MO diagram of **4**–**7** shown in Figure 5. The NO_2_-substituted complexes **4** and **5** have their frontier orbitals lower in energy, as compared to complexes **6** and **7** that bear electro-donor substituents on the O,N,O-tridentate ligand. Although they have somewhat different energies, the LUMO’s of the four complexes are of similar π*(pyridine) nature (Figure S10). Similarly, their HOMO’s can be approximated to the conjugated π HOMO of the dianionic O,N,O-Schiff base ligand, perturbed by the R^1^ and R^2^ substituents (Figure S10). The highest occupied level of substantial metallic character is the HOMO-2 or HOMO-3, depending on the compound (including **7**), suggesting that the metal will play a negligible role in most of their physico-chemical properties.

Shifting now to the dimer **8**, one can again notice the good match between the X-ray and optimized geometries (Figure S9 and Table 1). The computed C≡C bond distance (1.208 Å) agrees well with its experimental counterpart (1.199 Å). Its computed *ν*(C≡C) vibrational frequency (2350 cm^−1^) is slightly larger than that found for **5** (calc. 2235 cm^−1^; exp. 2227 cm^−1^, see above). Among the few (moderate) structural differences between the X-ray and DFT geometries, one can notice a lower planarity of the O,N,O-ligands in the optimized geometry and a more linear O1-Ni-O2 angle (176° *vs.* 167° in the X-ray structure, see Table 1 and discussion above). These differences are likely originating from the packing force that exist only in the solid state. Although both the metrical data (Table 1) and NAO charges (Table 2) of **8** are consistent with negligible Ni^…^Ni communication (i.e. two independent metal centers), its MO diagram is not the duplication of two diagrams of the type of the mononuclear complexes **4**–**7**, for example. Indeed, if the metals are not interacting, the two halves of the bipyridine ligand do interact through the C≡C bond, creating a low-lying LUMO, which becomes the low-lying LUMO of **8** (Figure S10). This orbital is the bonding combination between the out-of-plane π*(C≡C) orbital and π*(pyridine) orbitals of proper symmetry match. Calculations using the long-range corrected LC-wHPBE functional, supposed to better take into account conjugation in highly delocalized systems (see Computational Details) provided similar structural data (Table S4), MO shapes and level ordering, in agreement with the limited electronic communication through the metal centers.

## 3. Materials and Methods

### 3.1. Materials and Physical Measurements

Reactions were carried out under a dry argon atmosphere using standard Schlenk techniques. The solvents were dried and distilled according to standard procedures [57]. 4-Iodopyridine, 4-ethynylpyridine hydrochloride, 2-aminophenol, 2-amino-4-nitrophenol, 2,4-pentadione, triethylamine, potassium *tert*-butoxide, nickel(II) nitrate hexahydrate, copper iodide and *trans*-bis-triphenylphosphine palladium(II) dichloride were purchased from commercial sources and used without further purification. The proligands CH_3_-C(=O)CH=C(CH_3_)N(H)-C_6_H_3_-(4-NO_2_)-2-OH (**1**) [58,59], 4-MeO-C_6_H_4_-C(=O)C=C(CH_3_)N(H)-C_6_H_4_-2-OH (**2**) [49], and CpFe(C_5_H_4_)-C(=O)C=C(CH_3_)N(H)-C_6_H_4_-2-OH (**3**) [49], were synthesized according to published procedures. Solid-state FT-IR spectra were recorded on a Perkin-Elmer, Model Spectrum One, spectrophotometer with KBr disks in the 4000 to 400 cm^−1^ range. Electronic spectra were obtained using a Thermo Scientific, Helios Omega, spectrophotometer. ^1^H NMR spectra were measured on a Bruker AVANCE III 400 Instrument. All NMR spectra are reported in parts per million (ppm, δ) relative to tetramethylsilane (Me_4_Si), with the residual solvent proton resonance used as internal standards. Coupling constants (*J*) are reported in hertz (Hz), and integrations are reported as number of protons. The following abbreviations are used to describe peak patterns: br = broad, s = singlet, d = doublet, t = triplet, m = multiplet. Chemical shift assignments are given according to the atom numbering schemes depicted in Figure 1, Figure 2 and Figure 3 and Chart S1. Elemental analyses of **4–6** and **8** were conducted on a Thermo Finnigan Flash EA 1112 CHNS/O analyzer by the Microanalytical Service of the Centre Régional de Mesures Physiques de l’Ouest (Université de Rennes 1, France) and that of **7** was carried out with a Fisons - EA-1108 CHNS-O Element Analyzer (Thermo Scientific) by the Renewable Resources Laboratory of the Faculty of Chemical Sciences, UdeC (Chile). Melting points were determined in evacuated capillaries on a Kofler Bristoline melting point apparatus and were not corrected.

### 3.2. Synthesis of the Complexes

#### 3.2.1. [{CH_3_-C(O)CH=C(CH_3_)N-C_6_H_3_(4-NO_2_)-2-O}Ni(NC_5_H_4_I)] (**4**)

A Schlenk tube was charged with a magnetic stir bar, 248 mg (1.05 mmol) of proligand **1**, 354 mg (3.16 mmol) of potassium *tert*-butoxide and 1.0 mL of tetrahydrofuran (THF). After 5 min of stirring, a dark red precipitate formed and the reaction mixture was vigorously stirred for 25 min before adding 369 mg (1.8 mmol) of 4-iodopyridine. After 10 min of stirring, a solution of Ni(NO_3_)_2_·6H_2_O (437 mg, 1.5 mmol) in 5 mL of THF was added dropwise. The reaction mixture was vigorously stirred for 12 h at room temperature. The reaction was quenched with 10 mL of EtOH giving a precipitate. The solid material was filtered off, washed with 3 × 5 mL portion of cold (−5 °C) mixture of THF and EtOH (1:1, *v:v*), diethyl ether (2 × 3 mL portion), and dried under vacuum for 2 h. Yield: 366 mg (70%) of **4** as a light brown powder. Suitable monocrystals for X-ray diffraction study were obtained by slow evaporation (3 days) of a solution of the compound in dichloromethane petroleum ether mixture (2:1, *v:v*). m.p. 193 °C. Analysis calculated for C_16_H_14_IN_3_NiO_4_ (497.89 g mol^−1^): C, 38.60; H, 2.83; N, 8.44. Found: C, 38.37; H, 2.84; N, 8.50. FT-IR (KBr, cm^−1^): 3045(w) *ν*(C-H aryl), 2938(w) *ν*(C-H alkyl), 2828(w) *ν_sim_*(CH_3_), 1599(s) *ν*(C^…^O), *ν*(C^…^N) and/or *ν*(C^…^C), 1550(s), 1300(s) *ν_sym_*(N-O), 755(s) δ(C-H). ^1^H NMR (400 MHz, CD_2_Cl_2_): 1.84 (s, 3 H, O=C-CH_3_), 2.42 (s, 3 H, CH_3_), 5.27 (s, 1 H, H-9), 6.44 (d, ^3^*J*_H,H_ = 7.9 Hz, 1 H, H-6), 7.66 (s,1 H, H-3), 7.70 (d, ^3^*J*_H,H_ = 7.4 Hz, 1 H, H-5), 7.75 (br s, 2 H, H-13 and H-15), 8.08 (s, 2 H, H-12 and H-16).

#### 3.2.2. [{CH_3_-C(O)CH=C(CH_3_)N-C_6_H_3_(4-NO_2_)-2-O}Ni(4-NC_5_H_4_-C≡CH)] (**5**)

This compound was obtained following a procedure similar to that described above for compound **4**, using in this case, 248 mg (1.05 mmol) of the proligand **1**, 354 mg (3.16 mmol) of potassium *tert*-butoxide, 1.0 mL of THF, 140 mg (1.8 mmol) of 4-ethynylpyridine hydrochloride, and 437 mg (1.5 mmol) of Ni(NO_3_)_2_·6H_2_O in 5 mL of THF. Yield: 324 mg (78%) of a brown powder. Suitable monocrystals for X-ray diffraction were obtained by slow evaporation (4 days) of a solution of the compound in dichloromethane. m.p. 145 °C. Analysis calculated for C_18_H_15_N_3_NiO_4_ (396.02 g mol^−1^): C, 54.59; H, 3.82; N, 10.61. Found: C, 54.74; H, 4.02; N, 10.59. FT-IR (KBr, cm^−1^): 3049(w) *ν*(C-H aryl), 2932(w) *ν*(C-H alkyl), 2832(w) *ν_sim_*(CH_3_), 2227(w) *ν*(C≡C), 1604(s) *ν*(C^…^O), *ν*(C^…^N) and/or *ν*(C^…^C), 1557(s) *ν_asym_*(N-O), 1310(s) *ν_sym_*(N-O), 764(s) δ(C-H). ^1^H NMR (400 MHz, CD_2_Cl_2_): 1.85 (s, 3 H, O=C-CH_3_), 2.43 (s, 3 H, CH_3_), 3.49 (s, 1 H, C≡CH), 5.27 (s, 1 H, H-9), 6.47 (d, ^3^*J*_H,H_ = 7.6 Hz, 1 H, H-6), 7.36 (d, ^3^*J*_H,H_ = 7.4 Hz, 2 H, H-13 and H-15), 7.68 (d, ^3^*J*_H,H_ = 7.4 Hz, 1 H, H-5), 8.13 (s, 1 H, H-3), 8.42 (br s, 2 H, H-12 and H-16).

#### 3.2.3. [{4-MeO-C_6_H_4_-C(O)CH=C(CH_3_)N-C_6_H_4_-2-O}Ni(4-NC_5_H_4_I)] (**6**)

This compound was obtained following a procedure similar to that described above for compound **4**, using in this case, 297 mg (1.05 mmol) of the proligand **2**, 354 mg (3.16 mmol) of potassium *tert*-butoxide, 1.0 mL of THF, 369 mg (1.8 mmol) of 4-iodopyridine, and 437 mg (1.5 mmol) of Ni(NO_3_)_2_·6H_2_O in 1.5 mL of THF. Yield: 417 mg (73%) of dark red microcrystals. A monocrystal of this crop was selected and used for X-ray diffraction analysis. m.p. 190 °C. Analysis calculated for C_22_H_19_IN_2_NiO_3_ (544.99 g mol^−1^): C, 48.49; H, 3.51; N, 5.14. Found: C, 48.58; H, 3.47; N, 5.25. FT-IR (KBr, cm^−1^): 3049(w) *ν*(C-H aryl), 2932(w) *ν*(C-H alkyl), 2832(w) *ν_sym_*(CH_3_), 1604(s) *ν*(C^…^O), *ν*(C^…^N) and/or *ν*(C^…^C), 1557(s), 1244(s) *ν_asym_*(CH_3_-O-Ph), 764(s) δ(C-H). ^1^H NMR (400 MHz, CD_2_Cl_2_): 2.48 (s, 3 H, CH_3_), 3.72 (s, 3 H, OCH_3_), 5.83 (s, 1 H, H-9), 6.32 (t, ^3^*J*_H,H_ = 7.5 Hz, 1 H, H-4), 6.50 (d, ^3^*J*_H,H_ = 7.8 Hz, 1 H, H-6), 6.70 (t, ^3^*J*_H,H_ = 7.5 Hz, 1 H, H-5), 6.75 (d, ^3^*J*_H,H_ = 8.7 Hz, 2 H, H-13 and H-15), 7.23 (d, ^3^*J*_H,H_ = 8.2 Hz, 1 H, H-3), 7.46 (d, ^3^*J*_H,H_ = 8.5 Hz, 2 H, H-12 and H-16), 7.73 (br s, 2 H, H-19 and H-21), 8.24 (br s, 2 H, H-18 and H-22).

#### 3.2.4. [{CpFe(C_5_H_4_)-C(O)CH=C(CH_3_)N-C_6_H_4_-2-O}Ni(4-NC_5_H_4_I)] (**7**)

This organometallic derivative was obtained following a procedure similar to that described above for compound **4**, using in this case 383 mg (1.05 mmol) of the metalloligand **3**, 354 mg (3.16 mmol) of potassium *tert*-butoxide, 1.0 mL of THF, 369 mg (1.8 mmol) of 4-iodopyridine, and 437 mg (1.5 mmol) of Ni(NO_3_)_2_·6H_2_O in 1.5 mL of THF. Yield: 391 mg (60%) of a red powder. m.p. 188 °C. Analysis calculated for C_25_H_21_FeIN_2_NiO_2_ (622.89 g mol^−1^): C, 48.21; H, 3.40; N, 4.50. Found: C, 47.87; H, 3.86; N, 4.99. FT-IR (KBr, cm^−1^): 3049(w) *ν*(C-H aryl), 2932(w) *ν*(C-H alkyl), 2832(w) *ν_sym_*(CH_3_), 1604(s) *ν*(C^…^O), *ν*(C^…^N) and/or *ν*(C^…^C), 1557(s), 1244(s), 764(s) δ(C-H). ^1^H NMR (400 MHz, CD_2_Cl_2_): 2.43 (s, 3 H, CH_3_), 4.06 (s, 5 H, C_5_H_5_), 4.22 (br s, 2 H, H_β_ C_5_H_4_), 4.42 (br s, 2 H, H_α_ C_5_H_4_), 5.52 (s, 1 H, H-9), 6.31 (t, ^3^*J*_H,H_ = 7.5 Hz, 1 H, H-4), 6.50 (d, ^3^*J*_H,H_ = 7.8 Hz, 1 H, H-6), 6.67 (t, ^3^*J*_H,H_ = 7.4 Hz, 1 H, H-5), 7.21 (d, ^3^*J*_H,H_ = 8.0 Hz, 1 H, H-3), 7.77 (br s, 2 H, H-22 and H-24), 8.25 (br s, 2 H, H-21 and H-25).

#### 3.2.5. [{(4-MeO-C_6_H_4_-C(O)CH=C(CH_3_)N-C_6_H_4_-2-O)Ni}_2_(µ-NC_5_H_4_-C≡C-C_5_H_4_N)] (**8**)

A Schlenk tube was charged with a magnetic stir bar, 85 mg (0.12 mmol) of *trans*-PdCl_2_(PPh_3_)_2_ (20% mmol), 46 mg (0.24 mmol) of CuI (40% mmol), 50 mL of THF and 10 mL of triethylamine The reaction mixture was stirred for 5 min. Then, 300 mg (0.60 mmol) of complex **6** and 126 mg (0.90 mmol) of 4-ethynylpyridine hydrochloride were successively added. Argon was bubbled for 15 min into the solution, which was then stirred at room temperature for 12 h. The reaction mixture was filtered on celite and the filtrate was removed under vacuum to provide a dark red powder. The solid material was washed with a 5 × 5 mL portion of methanol dichloromethane mixture (3:1, *v:v*). Yield: 210 mg (0.24 mmol, 43%) of a red powder. Suitable monocrystals for X-ray diffraction study were obtained by slow evaporation (8 days) of a solution of the compound in dichloromethane. Analysis calculated for C_46_H_38_N_4_Ni_2_O_6_ (860.17 g mol^−1^): C, 64.23; H, 4.45; N, 6.51. Found: C, 64.58; H, 4.49; N, 6.38. FT-IR (KBr, cm^−1^): 3008(w) *ν*(C-H aryl), 2958(w) *ν*(C-H alkyl), 2825(w) *ν_sym_*(CH_3_), 2111(vw) *ν*(C≡C), 1604(s) *ν*(C^…^O), *ν*(C^…^N) and/or *ν*(C^…^C), 1558(s), 1244(s) *ν_asym_*(CH_3_-O-Ph), 728(s) δ(C-H). ^1^H NMR (400 MHz, CD_2_Cl_2_): 2.59 (s, 6 H, H-7 and H-7^1^), 3.83 (s, 6 H, H-17 and H-17^1^), 5.93 (s, 2 H, H-9 and H-9^1^), 6.44 (t, ^3^*J*_H,H_ = 7.2 Hz, 2 H, H-4 and H-4^1^), 6.66 (d, ^3^*J*_H,H_ = 7.7 Hz, 2 H, H-6 and H-6^1^), 6.80 (t, ^3^*J*_H,H_ = 7.3 Hz, 2 H, H-5 and H-5^1^), 6.86 (d, ^3^*J*_H,H_ = 8.8 Hz, 4 H, H-13, H13^1^ & H-15, H-15^1^), 7.39 (d, ^3^*J*_H,H_ = 8.1 Hz, 2 H, H-3 and H-3^1^), 7.49 (br s, 4 H, H-19, H-19^1^ and H-21, H-21^1^), 7.60 (d, ^3^*J*_H,H_ = 8.8 Hz, 4 H, H-12, H-12^1^ and H-16, H-16^1^), 8.73 (br s, 4 H, H-18 and H-22).

### 3.3. X-ray Crystal Structure Determinations

A crystal of appropriate size and shape of compounds **4**, **5**, **6**, **8** and **9** was coated in Paratone-N oil, mounted on a cryoloop and transferred to the cold gas stream of the cooling device. Intensity data were collected at T = 150(2) K on a Bruker APEXII Kappa-CCD diffractometer equipped with a bidimensional CCD plate detector, using Mo-Kα radiation (λ = 0.71073 Å), and were corrected for absorption effects using multiscanned reflections. The five structures were solved by dual-space algorithm using the SHELXT program [60], and then refined with full-matrix least-square method based on F^2^ (SHELXL-2014) [61]. In compound **8**, the two C_6_-aromatic rings exhibited a rotational disorder and have been modelled over two positions with equally refined occupancy of 0.50. The contribution of the very disordered dichloromethane crystallization solvent to the calculated structure factors of **9** were estimated following the BYPASS algorithm [62], implemented as the SQUEEZE option in PLATON [63]. Then, a new data set, free of solvent contribution, was used in the final refinement. All non-hydrogen atoms were refined with anisotropic atomic displacement parameters. Hydrogen atoms were placed in their geometrically idealized positions and constrained to ride on their parent atoms. A summary of the details about the data collection and refinement for the X-ray structures of the five compounds are documented in Table 3, and additional crystallographic details are in the CIF files. ORTEP views were drawn using OLEX2 software [64]. CCDC 2091893-2091897 (Table 3) contains the supplementary crystallographic data for this paper. These data can be obtained free of charge via http://www.ccdc.cam.ac.uk (accessed on 22 August 2021, or from the CCDC, 12 Union Road, Cambridge CB2 1EZ, UK; Fax: +44 1223 336033; E-mail: deposit@ccdc.cam.ac.uk).

### 3.4. Computational Details

DFT calculations [65,66,67] were carried out using the Gaussian16 package [68]. For the sake of consistency with our previous work [51], we employed the Def2TZVP basis set [69,70] together with the robust and rather computationally cheap PBE0 hybrid functional [71,72,73]. Since the investigated complexes possess conjugated fragments, the opportunity to use a long-range corrected functional [74,75,76] was also considered. Test calculations on complex **8** with the LC-wHPBE functional [77] provided similar structural data as with the PBE0 functional (Table S4), thus substantiating the use of the latter. The optimized geometries were fully characterized as true minima by analytical frequency calculations (no imaginary values). The geometries obtained from DFT calculations were used to perform natural atomic charge analysis with the NBO 6.0 program [78].

## 4. Conclusions

To summarize, five new four-coordinate nickel(II) complexes supported on O,N,O-tridentate Schiff base platforms have been synthesized and characterized using FT-IR and NMR spectroscopy, while their bulk purity was established through combustion analysis. The crystal structures of the three mononuclear species **4–6** and of the 4,4’-dipyridylethyne bridged bimetallic derivative **8** were elucidated by X-ray diffraction using single crystals, confirming (i) a square planar geometry at the Ni(II) center, (ii) the pincer-type nature of the Schiff base ligand that efficiently chelates the Ni(II) ions in a tridentate manner, and (iii) that the fourth coordination site is occupied by a functionalized pyridine. DFT calculations on complexes **4–7** indicate that the nature of the various substituents on the ligands has little effect on the nickel coordination sphere, but significantly perturb the energies of the HOMO and LUMO, which are ligand-based orbitals. The HOMO and LUMO being of pyridine and Schiff-base nature, respectively, this family of stable complexes is likely to display interesting NLO or photoluminescence properties that could be modulated by playing with the nature of the substituents. Although the two metal centers in **8** are not communicating, the electronic structure of **8** differs from that of the mononuclear species **4–7** by the existence of a bis(4-pyridyl) acetylene-based LUMO of low energy that led to expect different optical properties.

## Figures and Tables

**Table 1:** Selected bond distances (Å) and angles (°) for compounds **4**–**8**. Upper value: X-ray; lower value (in italics): DFT. Values into brackets are the corresponding computed Wiberg bond indices.

KERRYPNX	4	5	6	7 ^a^	8
Bond distances					
Ni(1)-O(1)	1.8365(14) *1.830* [0.368]	1.828(2) *1.831* [0.367]	1.819(3) *1.823* [0.388]	*1.824* [0.388]	1.766(6) ^b^, 1.892(5) ^b^ *1.823* [0.387]
Ni(1)-O(2)	1.8313(14) *1.831* [0.334]	1.817(2) *1.832* [0.334]	1.812(3) *1.834* [0.323]	*1.836* [0.322]	1.817(2) *1.833* [0.323]
Ni(1)-N(1)	1.8786(16) *1.877* [0.399]	1.880(2) *1.877* [0.397]	1.880(3) *1.874* [0.403]	*1.872* [0.404]	1.876(3) *1.873* [0.404]
Ni(1)-N(2)	1.9402(16) *1.954* [0.291]	1.920(2) *1.949* [0.295]	1.924(3) *1.951* [0.286]	*1.950* [0.286]	1.962(3) *1.951* [0.289]
Bond angles					
O(1)-Ni(1)-O(2)	176.53(6) *176*	174.92(9) *177*	173.55(13) *176*	*175*	167.39(19) *176*
N(1)-Ni(1)-N(2)	175.88(7) *175*	175.73(10) *175*	175.22(15) *175*	*176*	174.85(11) *175*
O(1)-Ni(1)-N(1)	87.39(7) *87*	87.44(9) *87*	88.34(14) *87*	*87*	86.8(2) *87*
O(1)-Ni(1)-N(2)	88.84(7) *89*	88.58(9) *89*	87.01(14) *88*	*88*	88.10(19) *88*
O(2)-Ni(1)-N(1)	95.96(7) *97*	97.60(9) *96*	97.72(14) *97*	*96*	97.18(10) *96*
O(2)-Ni(1)-N(2)	87.83(7) *88*	86.36(9) *88*	86.97(14) *88*	*88*	87.93(10) *88*

^a^ No X-ray structure data. ^b^ rotational disorder of the [C_6_H_4_O] fragment over two positions with equal occupancy (see Section 3.3 below).

**Table 2:** Computed NAO charges within the nickel coordination sphere.

	4	5	6	7	8
F	1.03	1.03	1.03	1.03	1.03
N(1)	−0.52	−0.52	−0.51	−0.51	−0.51
N(2)	−0.46	−0.45	−0.45	−0.45	−0.45
O(1)	−0.71	−0.71	−0.73	−0.73	−0.73
O(2)	−0.69	−0.69	−0.70	−0.70	−0.70

**Table 3:** Crystal data, details of data collection and structure refinement parameters for compounds **4**, **5**, **6**, **8** and **9**.

	4	5	6	8	9
Empirical Formula	C_16_H_14_IN_3_NiO_4_	C_18_H_15_N_3_NiO_4_	C_22_H_19_IN_2_NiO_3_	C_46_H_38_N_4_Ni_2_O_6_	C_36_H_30_I_2_P_2_Pd
Formula mass, g mol^−1^	497.91	396.04	545.00	860.22	884.74
Collection T, K	150(2)	150(2)	150(2)	150(2)	150(2)
crystal system	Monoclinic	Orthorhombic	Orthorhombic	Monoclinic	Monoclinic
space group	P2_1_/c	Pcnb	Pbca	P2_1_/c	C_2_/c
*a* (Å)	5.4337(4)	13.5799(16)	7.3551(4)	11.3760(15)	12.1810(5)
*b* (Å)	26.3384(17)	14.3565(17)	22.5317(16)	5.1862(6)	15.3526(6)
*c* (Å)	11.6425(8)	16.9584(18)	24.0937(16)	32.521(4)	19.4328(8)
*β (°)*	92.270(3)	90	90	90.782(6)	91.777(2)
*V* (Å^3^)	1664.9(2)	3306.2(7)	3992.9(4)	1918.5(4)	3632.4(3)
*Z*	4	8	8	2	4
*D*_calcd_ (g cm^−3^)	1.986	1.591	1.813	1.489	1.618
Crystal size (mm)	0.28 × 0.22 × 0.03	0.41 × 0.32 × 0.03	0.22 × 0.09 × 0.07	0.58 × 0.07 × 0.03	0.50 × 0.13 × 0.07
Crystal color	Red	Orange	orange	Brown	Red
Crystal description	Plate	Plate	Prism	Stick	Stick
*F*(000)	976	1632	2160	892	1712
abs coeff (mm^−1^)	3.047	1.204	2.545	1.039	2.321
*θ* range (°)	2.336–27.480	2.790–27.515	3.034–27.485	1.790–27.571	2.853–27.442
range h,k,l	−7/6, −34/30, −15/15	−17/11, −13/18, -21/21	−9/6, −21/29, −31/28	−14/10, −6/6, −42/42	−15/15, −19/16, −25/25
No. total refl.	26142	14085	19201	15866	28071
No. unique refl.	3791	3777	4572	4368	4140
R_int_	0.0319	0.0560	0.0784	0.0489	0.0336
Comp. *θ*_max_ (%)	99.7	99.1	99.8	98.6	99.7
Max/min transmission	0.913/0.711	0.965/0.634	0.837/0.663	0.969/0.740	0.850/0.598
Data/Restraints/Parameters	3791/0/228	3777/0/237	4572/0/264	4368/0/282	4140/0/186
Final R [*I* > 2*σ*(*I*)]	R_1_ = 0.0198 wR_2_ = 0.0449	R_1_ = 0.0399 wR_2_ = 0.0962	R_1_ = 0.0451 wR_2_ = 0.0874	R_1_ = 0.0523 wR_2_ = 0.1087	R_1_ = 0.0184 wR_2_ = 0.0507
R indices (all data)	R_1_ = 0.0225 wR_2_ = 0.0469	R_1_ = 0.0758 wR_2_ = 0.1146	R_1_ = 0.0705 wR_2_ = 0.0981	R_1_ = 0.0873 wR_2_ = 0.1248	R_1_ = 0.0206 wR_2_ = 0.0525
Goodness of fit/F^2^	1.137	1.028	1.032	0.974	0.890
Largest diff. Peak/hole (eÅ^−3^)	0.451/−0.529	0.592/−0.481	0.632/−0.800	0.419/−0.619	0.501/−0.421
CCDC number	2091896	2091897	2091893	2091894	2091895

## Data Availability

Not applicable.

## Supplementary Materials

The following are available online, Chart S1: Hydrogen and carbon atom labelling schemes for complex **7** used for NMR assignments, Figure S1: FT-IR spectrum of **8** recorded in KBr disk, Figure S2: ^1^H NMR spectrum of complex **7**, recorded at 400 MHz in CD_2_Cl_2_, Figure S3: ^1^H NMR spectrum of complex **8**, recorded at 400 MHz in CD_2_Cl_2_, Figure S4: Molecular structure of *trans*-PdI_2_(PPh_3_)_2_ (**9**), Figure S5: Crystal packing of **4** expanded through a-axis by H-bond interactions, Figure S6: Crystal packing of **5** expanded in the a- and c-axis plane by H-bond interactions, Figure S7: Crystal packing of **6** expanded in the a-axis direction, Figure S8: Crystal packing of **8** expanded in the b-axis through H-bond interactions, Figure S9: The DFT-optimized structures of complexes **4–6** and **8**, Figure S10: The HOMOs and LUMOs of complexes **4–8**, Table S1. UV-vis absorption data for compound **8** and its 4,4’-bipy bridged counterpart. Table S2: bond distances (Å) and angles (°) for compounds **4**, **5**, **6** and **8**, Table S3: Hydrogen bond interactions in **4**, **5** and **8**, Table S4. Optimized bond distances (Å) obtained for compound **8** with the PBE0 and LC-wHPBE functionals.

## Sample Availability

Samples of the compounds in this article are not available from the authors.
