# Supplementary material for: Nickel(II)-Based Building Blocks with Schiff Base Derivatives: Experimental Insights and DFT Calculations
Source: Molecules. 2021 Sep 1;26(17):5316. doi: 10.3390/molecules26175316 (PMC8434171; doi:10.3390/molecules26175316)
Supplement: Supplementary file 1 [file molecules-26-05316-s001.zip › molecules-1312907-supplementary.pdf]

Supplementary Materials

Nickel(II)-based building blocks with Schiff base derivatives: Experimental insights and DFT calculations

Néstor Novoa, Carolina Manzur, Thierry Roisnel, Samia Kahlal, Jean-Yves Saillard, David Carrillo, and Jean-René Hamon

Contents Page

Chart S1. Hydrogen and carbon atom labelling schemes for **7** used for NMR assignments. 2

Figure S1. FT-IR spectrum of **8** recorded in KBr disk. 2

Figure S2. ^{1}H NMR spectrum of complex **7** recorded at 400 MHz in CD_2Cl_2. 3

Figure S3. ^{1}H NMR spectrum of complex **8** recorded at 400 MHz in CD_2Cl_2. 3

Figure S4. Molecular structure of *trans*-$PdI_2(PPh_3)_2$ (**9**) with labelling scheme for selected atoms. 4

Figure S5. Crystal packing of **4** expanded through a-axis by H-bond interactions. 4

Figure S6. Crystal packing of **5** expanded in the a- and c-axis plane by H-bond interactions. 5

Figure S7. Crystal packing of **6** expanded in the a-axis direction. 5

Figure S8. Crystal packing of **8** expanded in the b-axis by H-bond interactions. 6

Figure S9. The DFT-optimized structures of complexes **4**-**6** and **8**. 7

Figure S10. The HOMOs and LUMOs of complexes **4**-**8**. 8

Table S1. UV-vis absorption data for compound **8** and its 4,4'-bipy bridged counterpart. 9

Table S2. Selected bond distances (Å) and angles (°) for compounds **4**, **5**, **6** and **8**. 9

Table S3. Hydrogen bond interactions in compounds **4**, **5** and **8**. 10

Table S4. Optimized bond distances (Å) obtained for compound **8** with the PBE0 and LC-wHPBE functionals. 11

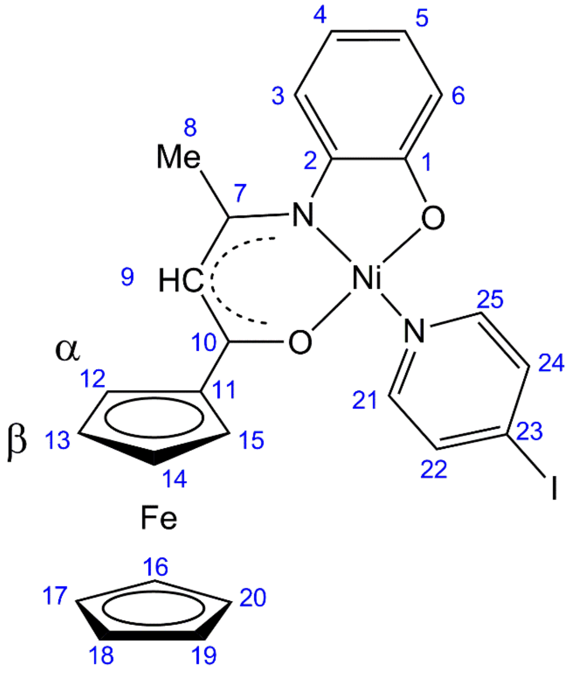

Chart S1. Hydrogen and carbon atom labelling schemes for complex **7** used for NMR assignments.

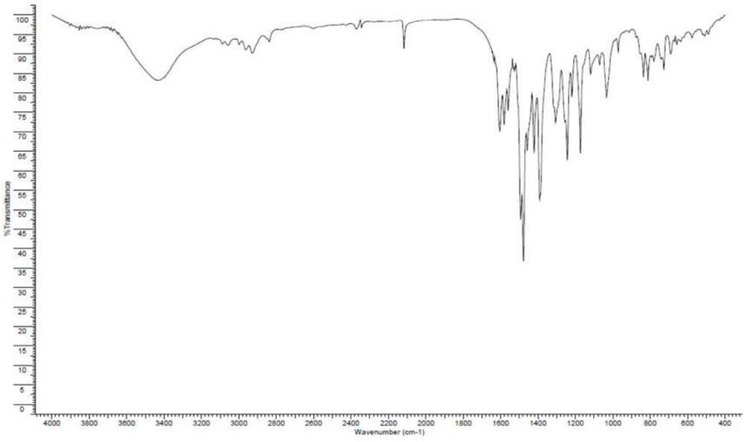

Figure S1. FT-IR spectrum of **8** recorded in KBr disk.

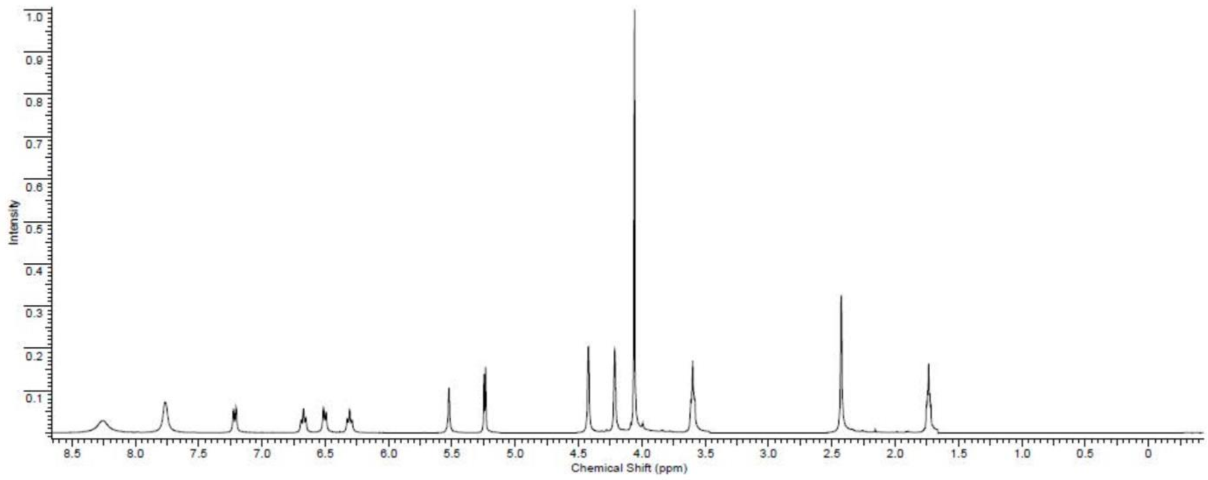

Figure S2. ^{1}H NMR spectrum of complex **7**, recorded at 400 MHz in CD_2Cl_2. See Chart S1 above for atom labelling scheme.

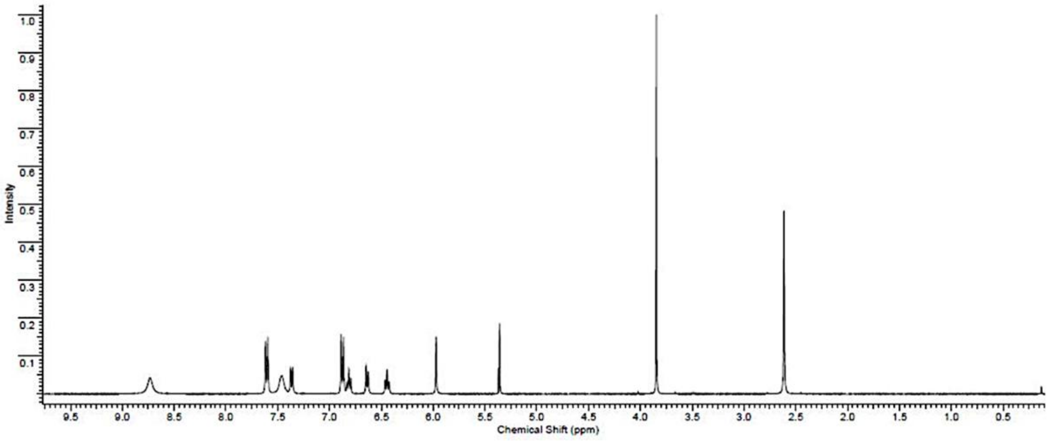

Figure S3. ^{1}H NMR spectrum of complex **8**, recorded at 400 MHz in CD_2Cl_2.

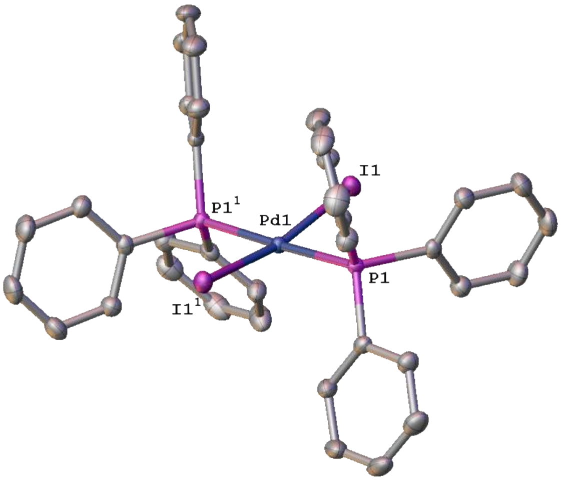

Figure S4. Molecular structure of *trans*-$PdI_2(PPh_3)_2$ (**9**) with labelling scheme for selected atoms. Hydrogens have been omitted for clarity. Thermal ellipsoids are drawn at the 50% probability level. Half unit of molecule was generated by symmetry operations #1 -x, -y, -z. Selected bond distances (Å) and angles (°): Pd(1)-I(1) = 2.61208(16), Pd(1)-P(1) = 2.3395(5); I(1)-Pd(1)-I(1)$^{\#1}$ = 168.226(11), p(1)-Pd(1)-P(1)$^{\#1}$ = 177.88(3), I(1)-Pd(1)-P(1) = 92.757(14), I(1)-Pd(1)-P(1)$^{\#1}$ = 87.024(13).

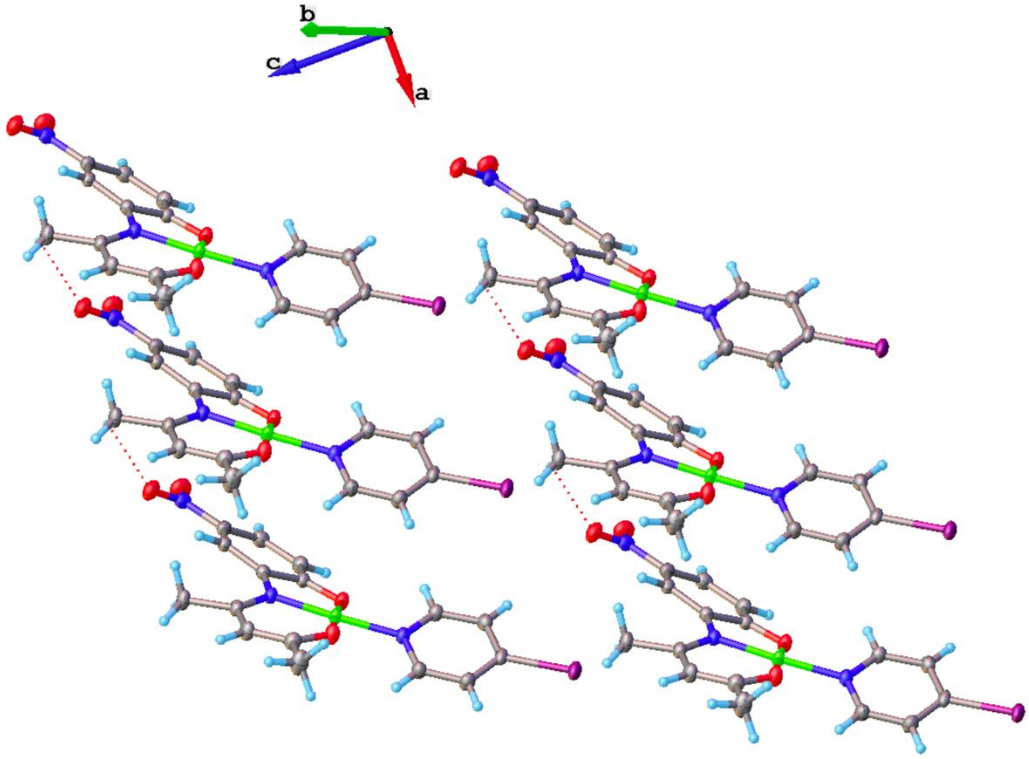

Figure S5. Crystal packing of **4** expanded through a-axis by H-bond interactions. Thermal ellipsoids are drawn at the 50% probability level.

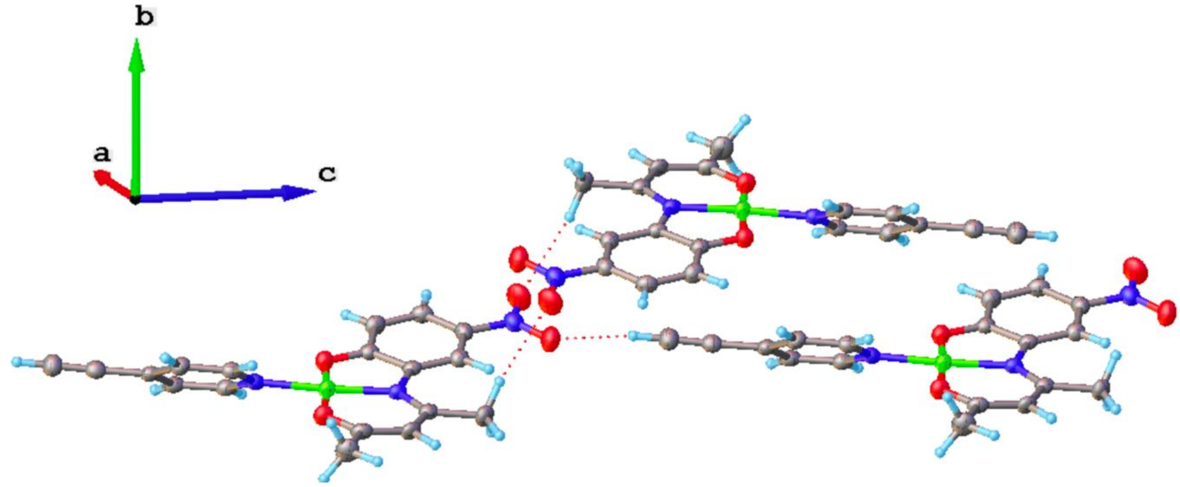

Figure S6. Crystal packing of **5** expanded in the a- and c-axis plane by H-bond interactions. Thermal ellipsoids are drawn at the 50% probability level.

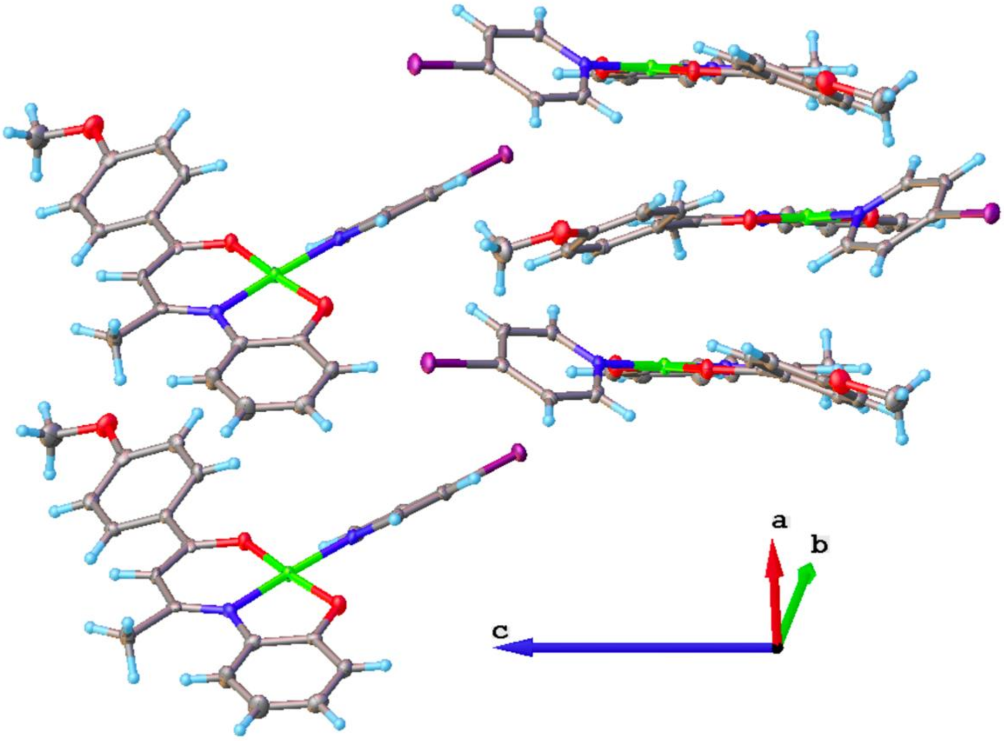

Figure S7. Crystal packing of **6** expanded in the a-axis direction, exhibiting a spiral alternate conformation of the molecules stabilized by weak interactions. Thermal ellipsoids are drawn at the 50% probability level.

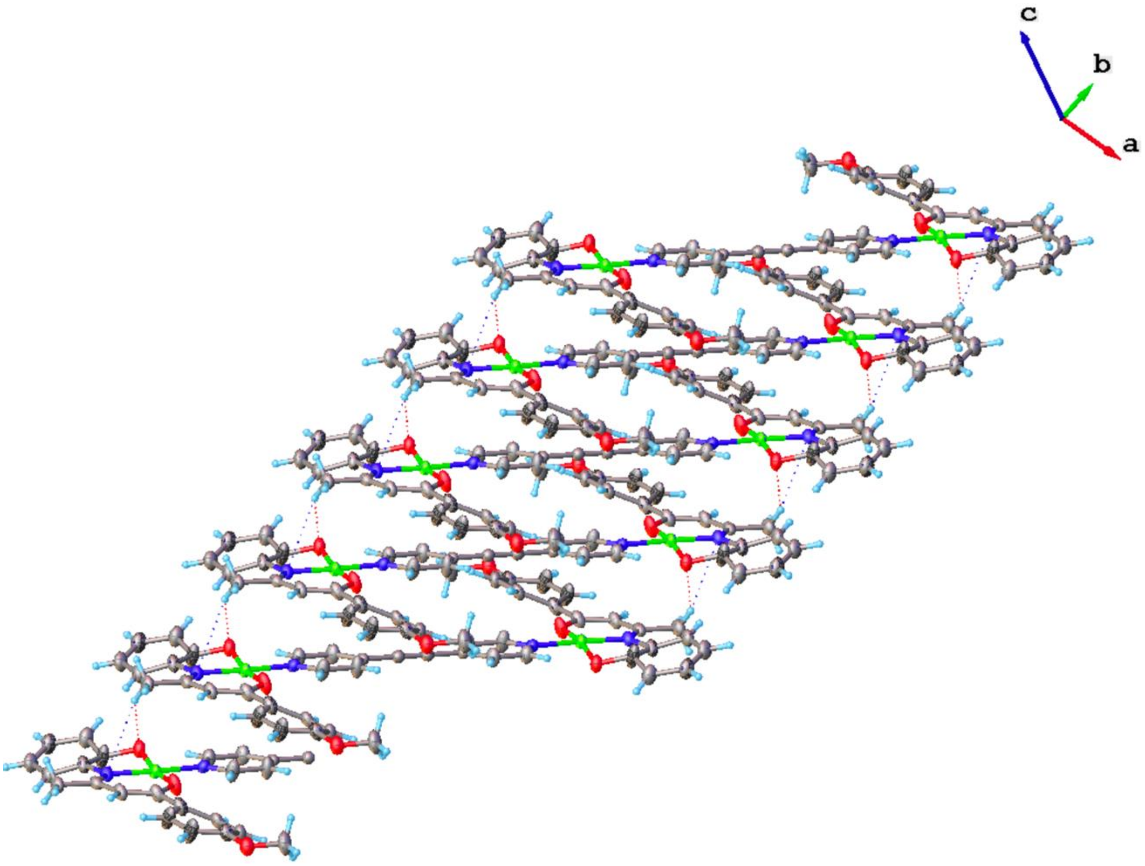

Figure S8. Crystal packing of **8** expanded in the b-axis through H-bond interactions. Thermal ellipsoids are drawn at the 50% probability level.

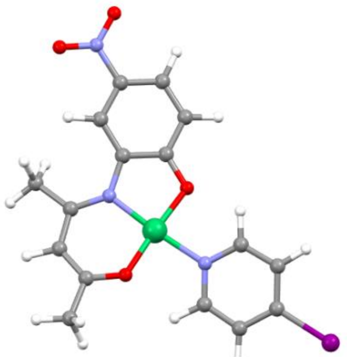

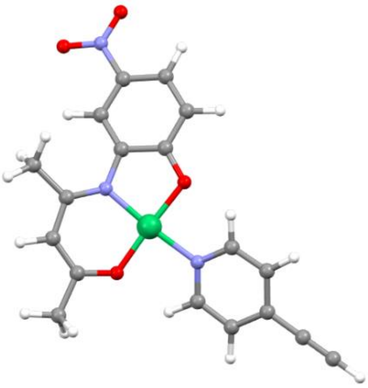

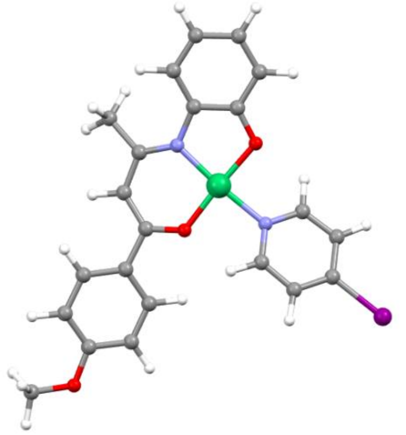

4 **5** **6**

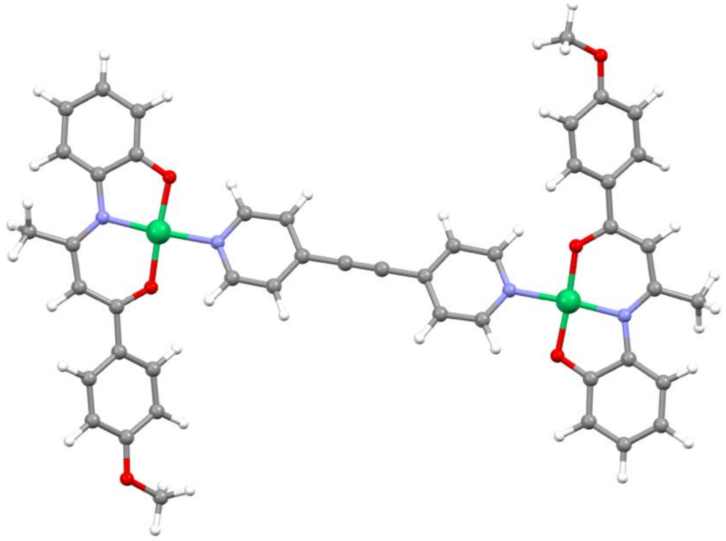

8

Figure S9. The DFT-optimized structures of complexes **4-6** and **8**.

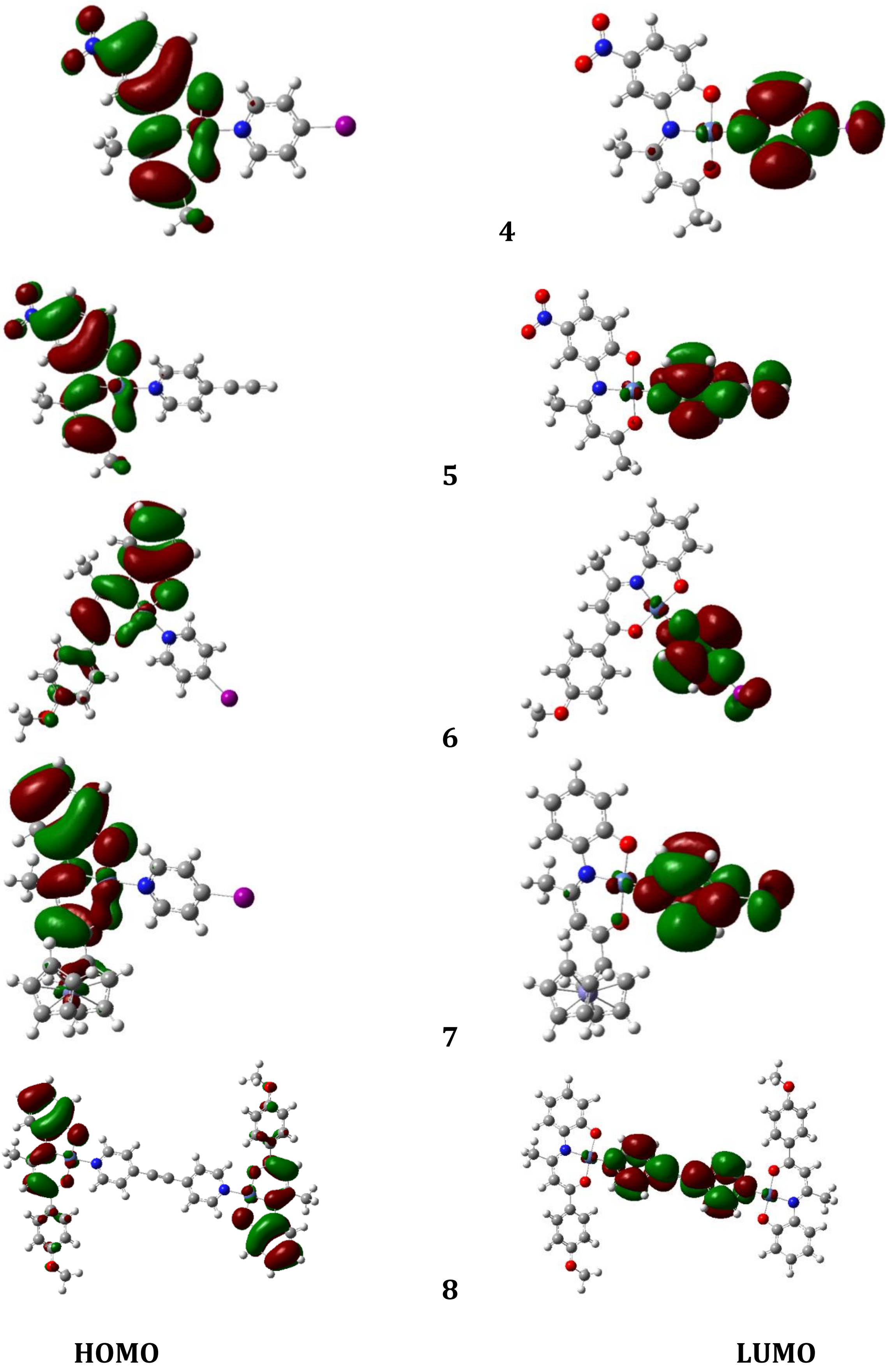

Figure S10. The HOMOs and LUMOs of complexes **4-8**.

Table S1. UV-vis absorption data for compound **8** and its 4,4’-bipy bridged counterpart.

Compd	Band	λ/nm (CH_2Cl_2)	Ref.
	a	295	
**8**	b	417	This work
	c	453	
	a	297	
**[{(An-ONO)Ni}$_2$(4,4’-vipy)]**	b	419	[44]
	c	456	

Table S2. Selected bond distances (Å) and angles (°) for compounds **4**, **5**, **6** and **8**.

	**4**	**5**	**6**	**8**
		Bond distances		
O(1)-C(1)	1.315(2)	1.309(3)	1.333(5)	1.424(7)
				1.340(7)
C(1)-C(2)	1.422(3)	1.419(4)	1.423(6)	1.361(5)
				1.361(5)
N(1)-C(2)	1.426(2)	1.426(3)	1.432(5)	1.441(4)
				1.441(4)
N(1)-C(8)	1.325(2)	1.330(4)	1.331(5)	1.327(4)
C(8)-C(9)	1.414(3)	1.408(4)	1.404(6)	1.410(5)
C(9)-C(10)	1.376(3)	1.371(4)	1.383(6)	1.376(5)
O(2)-C(10)	1.294(2)	1.296(3)	1.306(5)	1.314(4)
C(10)-C(11)	1.506(3)	1.507(4)	1.480(6)	1.487(5)
				1.487(5)
C^a-I(1)	2.0893(19)	-	2.096(4)	-
C^a-C^b	-	1.449(4)	-	1.442(4)
C^b≡C^c	-	1.159(4)	-	1.199(6)
O(3)-C(14)	-	-	1.365(5)	1.373(4)
				1.373(4)
O(3)-C(17)	-	-	1.424(5)	1.425(4)
N(3)-C(4)	1.448(2)	1.442(3)	-	-
N(3)-O(3)	1.238(2)	1.236(3)	-	-
N(3)-O(4)	1.237(2)	1.231(3)	-	-
		Bond angles		
Ni(1)-O(1)-C(1)	112.22(12)	112.55(18)	112.4(3)	111.1(4)
Ni(1)-O(2)-C(10)	124.78(13)	125.22(19)	126.4(3)	126.1(2)
Ni(1)-N(1)-C(2)	109.95(12)	110.07(17)	109.8(3)	110.22(19)
				110.22(19)
Ni(1)-N(1)-C(8)	123.98(13)	123.9(2)	123.3(3)	124.4(2)
O(1)-C(1)-C(2)	118.19(17)	118.3(3)	117.8(4)	114.0(4)
				118.5(4)
N(1)-C(2)-C(1)	111.08(16)	111.3(2)	111.6(4)	111.6(3)
				111.6(3)
N(1)-C(8)-C(9)	121.54(17)	121.9(3)	122.8(4)	122.3(3)
C(2)-N(1)-C(8)	125.12(16)	126.0(2)	126.9(4)	125.4(3)
				125.4(3)
O(2)-C(10)-C(9)	124.65(19)	124.7(3)	123.0(4)	123.6(3)
C(8)-C(9)-C(10)	125.73(19)	126.5(3)	126.7(4)	126.4(3)
C$_a$-C^b≡C^c	-	177.1(3)	-	179.2(4)
C(14)-O(3)-C(17)	-	-	118.1(4)	116.8(3)
				116.8(3)
O(3)-N(3)-O(4)	122.45(17)	121.3(3)	-	-
C(4)-N(3)-O(3)	119.09(18)	118.8(3)	-	-
C(4)-N(3)-O(4)	118.46(17)	119.9(3)	-	-

^{a} C14 for **4** and **5**, C20 for **6** and **8**. ^{b} C17 for **5**, C23 for **8**. *C*18 for **5**, C23$^{\#1}$ for **8** (#1 -x, -y, -z).

Table S3. Hydrogen bond interactions in **4**, **5** and **8**.

Compd.	D−H···A	D−H (Å)	H···A (Å)	D···A (Å)	D−H···A (°)
**4**	C6-H6…O1[#1]	0.95	2.61	3.538(2)	164.1
	C11-H11…O4[#2]	0.98	2.61	3.339(3)	131.1
**5**	C7-H7…O4[#3]	0.98	2.53	3.500(4)	168.8
	C12-H12…O4[#4]	0.95	2.54	3.359(4)	144.6
**8**	C3A-H3A…O1A[#5]	0.95	2.34	2.846(10)	112.7
	C6B-H6B…O1B[#6]	0.95	2.59	3.480(9)	157.0
	C7-H7B…O3[#7]	0.98	2.59	3.414(4)	142.0
	C7-H7C…O1A[#5]	0.98	2.61	3.482(6)	147.8
	C18-H18…O1A	0.95	2.16	2.684(7)	113.1
	C18-H18…O1B	0.95	2.16	2.732(6)	117.7
	C22-H22…O2	0.95	2.17	2.716(4)	115.3

Symmetry transformations used to generate equivalent atoms: #1 -x, -y, -z; #2 x, -y-1/2, z-1/2; #3 -x, -y, -z; #4 x+1/2, -y, -z+1/2; #5 x, y, z; #6 -x, -y, -z; #7 -x, y+1/2, -z+1/2.

Table S4. Optimized bond distances (Å) obtained for compound **8** with the PBE0 and LC-wHPBE functionals.

Distance	PBE0	LC-wHPBE
Ni1-N1	1.873	1.859
Ni1-N2	1.951	1.944
Ni1-O1	1.823	1.818
Ni1-O2	1.833	1.831
N2- C18	1.337	1.332
C18- C19	1.379	1.376
C19- C20	1.396	1.387
C20- C21	1.396	1.388
C21- C22	1.379	1.375
N2- C22	1.337	1.332
C20- C23	1.417	1.430
C23- C23^1	1.208	1.197
C23^1- C20^1	1.417	1.430
N2^1- C18^1	1.337	1.332
C18^1- C19^1	1.379	1.376
C19^1- C20^1	1.396	1.387
C20^1- C21^1	1.396	1.388
C21^1- C22^1	1.379	1.375
N2^1- C22^1	1.337	1.332
Ni1^1- N1^1	1.873	1.860
Ni1^1- N2^1	1.950	1.944
Ni1^1- O1^1	1.823	1.818
Ni1^1- O2^1	1.833	1.831
